# The First Structure of Human MTHFD2L and Its Implications for the Development of Isoform‐Selective Inhibitors

**DOI:** 10.1002/cmdc.202200274

**Published:** 2022-07-06

**Authors:** Emma R. Scaletti, Robert Gustafsson Westergren, Yasmin Andersson, Elisee Wiita, Martin Henriksson, Evert J. Homan, Ann‐Sofie Jemth, Thomas Helleday, Pål Stenmark

**Affiliations:** ^1^ Department of Biochemistry and Biophysics Stockholm University Svante Arrhenius väg 16 C Stockholm 106 91 Sweden; ^2^ Drug Discovery and Development Platform, Science for Life Laboratory School of Biotechnology Royal Institute of Technology Tomtebodavägen 23a Stockholm 17165 Sweden; ^3^ Science for Life Laboratory Department of Oncology-Pathology Karolinska Institute Tomtebodavägen 23a Stockholm 171 65 Sweden; ^4^ Department of Oncology and Metabolism The University of Sheffield Beech Hill Road Sheffield S10 2RX UK

**Keywords:** structural biology, cancer, enzyme inhibition, 1C-metabolism, methylenetetrahydrofolate dehydrogenase

## Abstract

Methylenetetrahydrofolate dehydrogenase 2 (MTHFD2) is a mitochondrial 1‐carbon metabolism enzyme, which is an attractive anticancer drug target as it is highly upregulated in cancer but is not expressed in healthy adult cells. Selective MTHFD2 inhibitors could therefore offer reduced side‐effects during treatment, which are common with antifolate drugs that target other 1C‐metabolism enzymes. This task is challenging however, as MTHFD2 shares high sequence identity with the constitutively expressed isozymes cytosolic MTHFD1 and mitochondrial MTHFD2L. In fact, one of the most potent MTHFD2 inhibitors reported to date, TH7299, is actually more active against MTHFD1 and MTHFD2L. While structures of MTHFD2 and MTHFD1 exist, no MTHFD2L structures are available. We determined the first structure of MTHFD2L and its complex with TH7299, which reveals the structural basis for its highly potent MTHFD2L inhibition. Detailed analysis of the MTHFD2L structure presented here clearly highlights the challenges associated with developing truly isoform‐selective MTHFD2 inhibitors.

## Graphical abstract

**Pinning down selectivity**: MTHFD2 is an anti‐cancer drug target only expressed in cancer cells. However, MTHFD2 inhibitors also target MTHFD1 and MTHFD2L, which are expressed in healthy cells. The first structure of human MTHFD2L and its complex with a potent inhibitor reveals the structural basis for inhibition differences between isoforms and highlights the challenges and opportunities in developing selective MTHFD2 inhibitors targeting the folate, cofactor and allosteric binding sites.

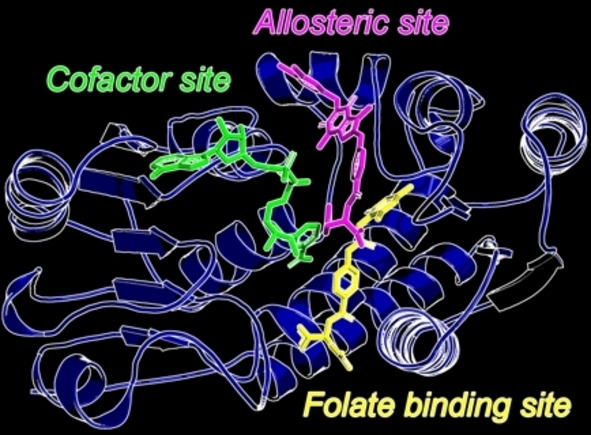

## Introduction

**Figure 1:**
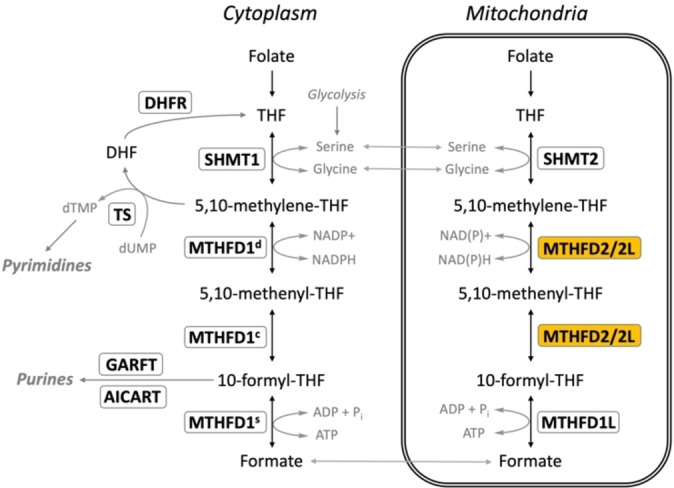
Overview of mammalian 1C‐metabolism. Overexpression of MTHFD2 and the other enzymes involved in folate metabolism is a feature of various cancers. Schematic representation of the enzymes involved in 1C‐metabolism, indicating their substrates and cellular localization within either the cytosol or mitochondria. THF (tetrahydrofolate), DHF (dihydrofolate). Enzymes are outlined in boxes: thymidylate synthetase (TS), dihydrofolate reductase (DHFR), β‐glycinamide ribonucleotide transformylase (GARFT), 5′‐amino‐4′‐imidazolecarboxamide ribonucleotide transformylase (AICARFT), cytosolic serine hydroxymethyltransferase (SHMT1), mitochondrial serine hydroxymethyltransferase (SHMT2), mitochondrial C1‐tetrahydrofolate synthase (MTHFD1L), methylene tetrahydrofolate dehydrogenase 1 (MTHFD1), methylenetetrahydrofolate dehydrogenase 2 (MTHFD2), methylene tetrahydrofolate 2‐like (MTHFD2L). Dehydrogenase activity (d), cyclohydrolase activity (c), synthetase activity (s).

The *de novo* synthesis of both purine and pyrimidine nucleotides is dependent on active one‐carbon (1C) units derived from folate that are generated through the 1C‐metabolism pathway. In eukaryotes 1C‐metabolism is highly compartmentalized between the cytoplasm and the mitochondria in the cell,[^1^, ^2^, ^3^] with the majority of 1C‐units used in the cytoplasm being derived from the mitochondria.[^4^, ^5^, ^6^, ^7^] Due to the importance of 1C‐metabolism in the production of DNA precursors it is unsurprising that the entire pathway is upregulated in cancer cells^[8]^ as well as in embryonic cells^[9]^ to sustain their rapid growth.[^10^, ^11^] In mitochondria, 1C‐units derived from serine by serine hydroxymethyltransferase (SHMT) are attached to tetrahydrofolate (THF), resulting in methylene−THF (CH2−THF) which is subsequently oxidized to formate. The formate then crosses the mitochondrial membrane to the cytosol, where it can be attached to THF again, resulting in 10‐formyl−THF that is used for *de novo* purine synthesis or is reduced further to 5,10‐methylene−THF, which is utilized for thymidylate or methionine synthesis. The different redox environments of the cytoplasm and mitochondria favor the production of 5,10‐methylene−THF in the cytoplasm and the production of formate in the mitochondria (Figure 1).[^2^, ^12^]

The methylene tetrahydrofolate dehydrogenase (MTHFD) family of proteins are responsible for the conversion between methylene−THF and formate, a process which involves three distinct enzymatic activities: (1) 5,10‐methylene−THF (CH2−THF) dehydrogenase, (2) 5,10‐methenyl−THF (CH^+^‐THF) cyclohydrolase and (3) 10‐formyl‐THF (10‐CHO−THF) synthetase.[^13^, ^14^] In the cytosol all three enzymatic functions are carried out by MTHFD1, which is expressed in all adult tissues examined to date.^[15]^ MTHFD1 consists of two distinct domains, each of which form homodimers. This involves the (1) DC domain, which performs the dehydrogenase (d) and cyclohydrolase (c) activities utilizing the same active site, with the dehydrogenase function requiring NADP^+^ as a cofactor[^16^, ^17^, ^18^] and (2) Synthetase domain, responsible for 10‐formyl synthetase (s) activity, which requires ADP and phosphate for activity (Figure 1).^[15]^ In the mitochondria, the interconversion of 5,10‐methylene−THF and formate is carried out by three enzymes: MTHFD1L, MTHFD2 and MTHFD2L.[^12^, ^19^] MTHFD1L is a homologue of cytosolic trifunctional MTHFD1,[^12^, ^20^, ^21^] however differences in residues responsible for the dehydrogenase and cyclohydrolase activities mean that the enzyme is effectively a monofunctional synthetase, only catalyzing the interconversion of 10‐formyl−THF and folate (Figure 1).[^20^, ^22^, ^23^] MTHFD1L is expressed in all embryonic and adult tissues examined to date.[^20^, ^24^]

The dehydrogenase and cyclohydrolase activities in the mitochondria are performed by MTHFD2 and the ‘MTHFD2‐like’ isoform MTHFD2L.^[19]^ MTHFD2 catalyzes the oxidation of methylene−THF to 10‐formyl−THF (Figure 1). MTHFD2 is proposed to have evolved from tri‐functional MTHFD1 through the loss of the synthetase domain and change to NAD^+^ (with an absolute requirement for P_i_ and Mg^2+^) as a cofactor instead of NADP^+^.[^22^, ^25^, ^26^, ^27^, ^28^] MTHFD2 displays some activity with NADP^+^, albeit much lower, and with this cofactor the enzyme only requires Mg^2+^, and not P_i_.[^27^, ^29^] *MTHFD2* gene inactivation has been shown to be embryonic lethal in mice.^[10]^ In fact, MTHFD2 is primarily expressed during embryonic development and is not significantly expressed in healthy adult cells.[^10^, ^11^, ^21^, ^24^, ^25^, ^26^, ^28^, ^30^, ^31^, ^32^] However, there is clear evidence of MTHFD2 upregulation and overexpression in cancer cells.[^33^, ^34^, ^35^, ^36^] During the last five years MTHFD2 has emerged as a highly relevant disease‐selective anticancer target, with selective inhibitors having the potential to eliminate cancer cells while sparing healthy cells.[^37^, ^38^, ^39^, ^40^, ^41^]

MTHFD2L is an isoform of MTHFD2 (72 % amino acid sequence identity), which also catalyzes oxidation of methylene−THF to 10‐formyl−THF (Figure 1).^[19]^ The enzyme is expressed in all adult tissues analyzed to date,^[19]^ as well as in embryonic cells.^[32]^ As MTHFD2 is not expressed in differentiated adult cells,[^11^, ^24^] MTHFD2L therefore fills the gap left in adult mammalian 1C‐metabolism left by the lack of dehydrogenase/cyclohydrolase activity in the monofunctional MTHFD1L enzyme.^[19]^ MTHFD2L is capable of using either NAD^+^ or NADP^+^ as a cofactor, however its overall catalytic efficiency is much lower than MTHFD2.[^19^, ^32^] At high concentrations of 5,10‐methylene−THF MTHFD2L prefers NAD^+^ as a cofactor. However, at concentrations below 10 μM the enzyme is more active with NADP^+^. The mitochondrial matrix levels of 5,10‐methylene−THF are estimated to range from 2.5 to 25 μM,[^42^, ^43^, ^44^, ^45^] suggesting that MTHFD2L likely exhibits dual redox cofactor specificity *in vivo*. The dual cofactor usage would allow MTHFD2L to quickly adapt to changing metabolic conditions. Studies of MTHFD2L isolated from rat liver mitochondria show that the enzyme behaves as a tightly associated peripheral membrane protein, which is located on the matrix side of the inner mitochondrial membrane.^[19]^ In contrast to MTHFD2, MTHFD2L is not upregulated in cancer,^[46]^ nor does it show an association with growth factor stimulation.[^34^, ^36^, ^47^] Importantly, MTHFD2L does not display a compensatory increase in expression when MTHFD2 is inhibited^[46]^ making it unlikely that the isozyme would be involved in the development of chemoresistance to such inhibitors. It has been proposed that MTHFD2L functions to maintain a baseline activity of mitochondrial folate metabolism, while MTHFD2 is upregulated in conditions where greater flux through the pathway is required, such as during embryogenesis or in cancer cells.[^29^, ^32^]

We previously determined the first structure of human MTHFD2 bound to the low micro‐molar folate analogue inhibitor LY3458999^[37]^ and we recently published crystal structures of MTHFD2 bound with the most potent nanomolar MTHFD2 inhibitors reported to date, which suppress acute myeloid leukemia (AML) through thymidine depletion and subsequent uracil misincorporation into DNA, which results in replication stress and apoptosis.^[41]^ It was noted that while these inhibitors do not target other enzymes in the 1C‐metabolism pathway, which is a limitation of current antifolate drugs in clinical use, they are potent inhibitors of all MTHFD isoforms. The inhibitor TH7299 for example, was shown to be even more potent towards MTHFD1 and MTHFD2L than MTHFD2.^[41]^ As MTHFD2L and MTHFD1 are expressed in healthy cells this may be of concern. However, this observation is unsurprising due to the high amino acid sequence identity shared by MTHFD proteins. Other less potent inhibitors have also been reported which target either the folate binding site[^39^, ^48^] or a recently identified allosteric site.^[40]^ Several structures of the MTHFD1 DC domain (MTHFD1−DC) bound with antifolate inhibitors have also been published.[^13^, ^18^] A limitation of other MTHFD2 inhibitor studies, is that when certain inhibitors are referred to as being ‘isoform‐selective’ it is only in the context of MTHFD2 selectivity against MTHFD1, and MTHFD2L inhibition is not tested.[^39^, ^40^] This is problematic as the majority of 1C‐units used in the cytoplasm are actually produced in the mitochondria,[^4^, ^6^] where MTHFD2L produces 1C‐units in healthy cells. It is therefore clear that in regard to developing more selective MTHFD2 inhibitors, that the inhibition of MTHFD2L in addition to MTHFD1 is a very important consideration.

We present the first structure of human MTHFD2L and its complex with our previously reported MTHFD2 inhibitor, TH7299. We performed inhibition analysis of the protein used for co‐crystallization, the results of which were consistent with our previous MTHFD2L inhibition data. The stronger inhibition of TH7299 toward MTHFD2L over MTHFD2 is clearly explained by our structural data which shows that while the folate binding sites are almost identical, there is a key arginine vs tyrosine difference. We propose this to be an important factor for the enhanced inhibition of TH7299 towards MTHFD2L, and as additional structural comparisons indicate, also MTHFD1. Detailed comparisons of our MTHFD2L structure with MTHFD2 reveals that the majority of the differences between the isoforms are located in surface exposed areas rather than in the folate binding site, cofactor binding site or the allosteric site, where any differences are predominantly physicochemically conserved changes. The dimer interface of MTHFD2L and MTHFD2 is also highly conserved. In contrast, structural similarities between MTHFD2L and MTHFD1 in terms of the three ligand binding sites and the dimer interface is significantly lower. Overall, these results clearly emphasize the challenges associated with the development of truly isoform‐selective MTHFD2 inhibitors.

## Results and Discussion

### Inhibition of MTHFD2L by TH7299

**Figure 2:**
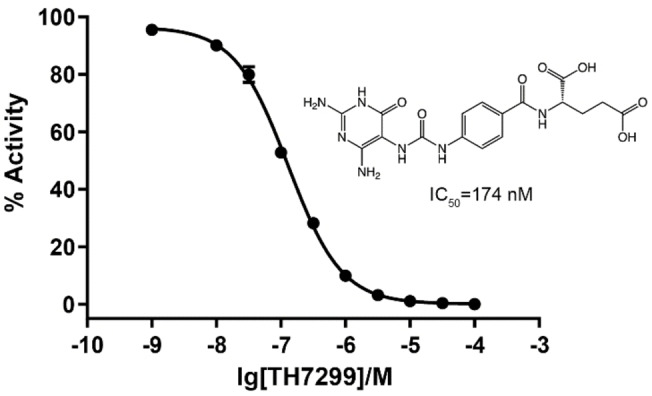
TH7299 dose‐response curve for MTHFD2L. IC_50_=174 nM (*n*=3).

Human MTHFD2L lacking the mitochondrial signal peptide was expressed in *E. coli* and purified to homogeneity via Immobilized metal affinity (IMAC) and Size‐exclusion (SEC) chromatography (refer to Experimental Section). We performed a TH7299 dose‐response curve for purified MTHFD2L which indicated an IC_50_ value of 174 nM (Figure 2), which is similar to our earlier reported IC_50_ value of 126 nM.^[41]^ TH7299 was originally developed by simplifying the tricyclic core of LY345899,^[41]^ which was first described as 9L9, an inhibitor of bacterial FolD.^[49]^ We previously solved the structure of MTHFD2 in complex with LY345899 and performed inhibition studies that showed it to be significantly more potent against cytoplasmic MTHFD1−DC (IC_50_: 96 nM) than MTHFD2 (IC_50_: 663 nM).^[37]^ As noted previously, while TH7299 is highly selective towards MTHFD2 (IC_50_: 254 nM) over other enzymes involved in 1C‐metabolism (i. e. SHMT1/2, TS and DHFR) it was also shown to be a more potent inhibitor of the structurally related MTHFD2 isoforms MTHFD2L (IC_50_: 126 nM) and MTHFD1−DC (IC_50_: 89 nM).^[41]^ As both MTHFD2L and MTHFD1−DC are expressed in healthy cells it would clearly be advantageous to develop inhibitors that are truly selective towards MTHFD2 to minimize potential side‐effects, a challenging task considering the high degree of sequence similarity between MTHFD isoforms. As no structural information existed prior to the current study, we aimed to solve the X‐ray crystal structure of MTHFD2L in complex with TH7299 to gain insight into the structural reasons for the significant differences in inhibition observed between MTHFD isoforms.

### Overall structure of human MTHFD2L

**Figure 3:**
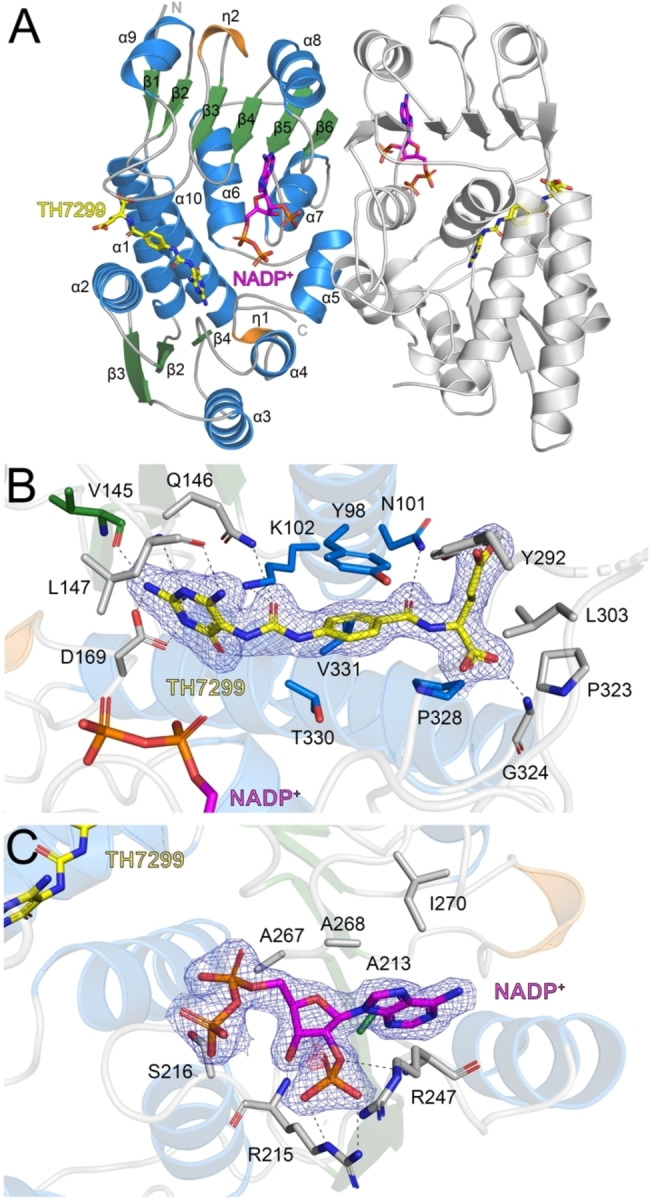
The MTHFD2L crystal structure. A) Structure of the hMTHFD2L dimer. The alpha‐helices (α1–10), beta‐strands (β1–9) and 3_10_‐helices (η1–2) of the first monomer are colored blue, green and orange, respectively. The second monomer is colored light grey. The N‐terminus (N) and C‐terminus (C) of the first monomer is labelled. The TH7299 inhibitor and NADP^+^ cofactor ligands are depicted as sticks; C atoms colored yellow (TH7299) or magenta (NADP^+^), O atoms red, N atoms dark blue and P atoms orange. Hydrogen bond networks for B) TH7299 in the folate binding site and C) Partial NADP^+^ in the co‐factor binding site of MTHFD2L. Hydrogen bond interactions are shown as dashed lines. The 2*F*_o_‐*F*_c_ electron density maps around TH7299 and NADP^+^ (panels B and C) are contoured at 1.0 σ (blue) and the *F*_o_‐*F*_c_ electron density maps are contoured at −3.0 σ (red) and +3.0 σ (green). Figures were produced with PyMOL (v.2.3.3, Schrödinger).

Following purification it was evident that MTHFD2L was highly unstable, consistent with our previous work^[41]^ and crystallization was only possible in the presence of a potent inhibitor. MTHFD2L was co‐crystallized with TH7299 and the inhibitor‐bound structure was solved to 2.10 Å resolution (Table 1). The protein crystallizes as a monomer in the asymmetric unit but is dimeric in solution. This agrees with studies of MTHFD2 and MTHFD1−DC, which are also dimers in solution.[^13^, ^18^, ^26^, ^37^] There is one small area of missing electron density in the structure, a disordered loop region corresponding to residues 294–301. The MTHFD2L monomer is comprised of two domains, which are connected via two long alpha helices (α1 and α10, Figure 3A) and a smaller alpha helix (α5, Figure 3A), positioned such that there is a large cleft between the domains. Analysis of the MTHFD2L dimer interface using PISA (Protein Interfaces, Surfaces and Assemblies)^[50]^ indicates a buried surface area of 1625 Å^2^.

**Table 1:** Data collection and refinement statistics for hMTHFD2L−TH7299.

Data Collection	
Space group	C222_1_
Cell dimensions:	
*a*, *b*, *c* [Å]	77.11, 109.26, 73.03
α, β, γ [°]	90, 90, 90
No. unique reflections	18319 (1478)
Resolution	47.70–2.10 (2.16–2.10)
*R*_merge_ [%]	30.1 (140.7)
*R*_pim_ [%]	11.8 (57.9)
mean (I/σI)	6.7 (1.4)
Completeness [%]	99.9 (99.5)
CC(1/2) [%]	98.5 (41.3)
Redundancy	7.3 (6.8)
Refinement Statistics	
*R*_work_/*R*_free_	18.7/23.1
*B‐*factors [A^2^]	
Protein	22.90
Ligand (TH7299)	20.80
Water	26.91
RMSD bond length [A]	0.012
RMSD bond angle [°]	1.52

### Folate and cofactor binding sites

There was clear electron density for the inhibitor TH7299 in the folate binding site (Figure 3B). The ligand is located in a cleft between the N‐ and C‐terminal domains, with the inhibitor primarily supported by residues from the N‐terminal domain (Figure 3A). The diaminopyrimidine head group of TH7299 is positioned by key hydrogen bond interactions with Asp169 and the main chain atoms of Val145 and Leu147. The urea linker and central phenyl ring are oriented by hydrogen bonding with Asn101, Lys102 and Gln146, as well as an important pi‐stacking interaction with Tyr98. The glutamate tail of the inhibitor forms hydrogen bonds with the backbone nitrogen of Gly324 and the side chain oxygen of Tyr292. TH7299 is additionally supported by hydrophobic interactions with Leu303, Pro232, Pro328, Thr330 and Val331 (Figure 3B).

NADP^+^ is bound within a large cleft formed between the N‐ and C‐terminal domains of MTHFD2L, with the cofactor mostly supported by interactions with the C‐terminal domain (Figure 3A). It was evident during structure refinement that there was only consistent electron density for part of NADP^+^, and the ribose and nicotinamide moiety at one end of the cofactor were therefore not included in the final structure (Figure 3C). This indicates that the cofactor may have been hydrolyzed during co‐crystallization. Within the cofactor binding site NADP^+^ is supported by hydrogen bond interactions between oxygen atoms of one of its phosphates and the main chain and side chain nitrogen atoms of Arg215 and Arg247, the former of which also hydrogen bonds with the 2′‐OH group of the sugar. The cofactor is also supported by extensive hydrophobic interactions with the residues Ala213, Gly214, Ser216, His246, Ala267, Ala268 and Ile270 (Figure 3C).

### Comparison of MTHFD2L with related isoforms: folate binding site

**Figure 4:**
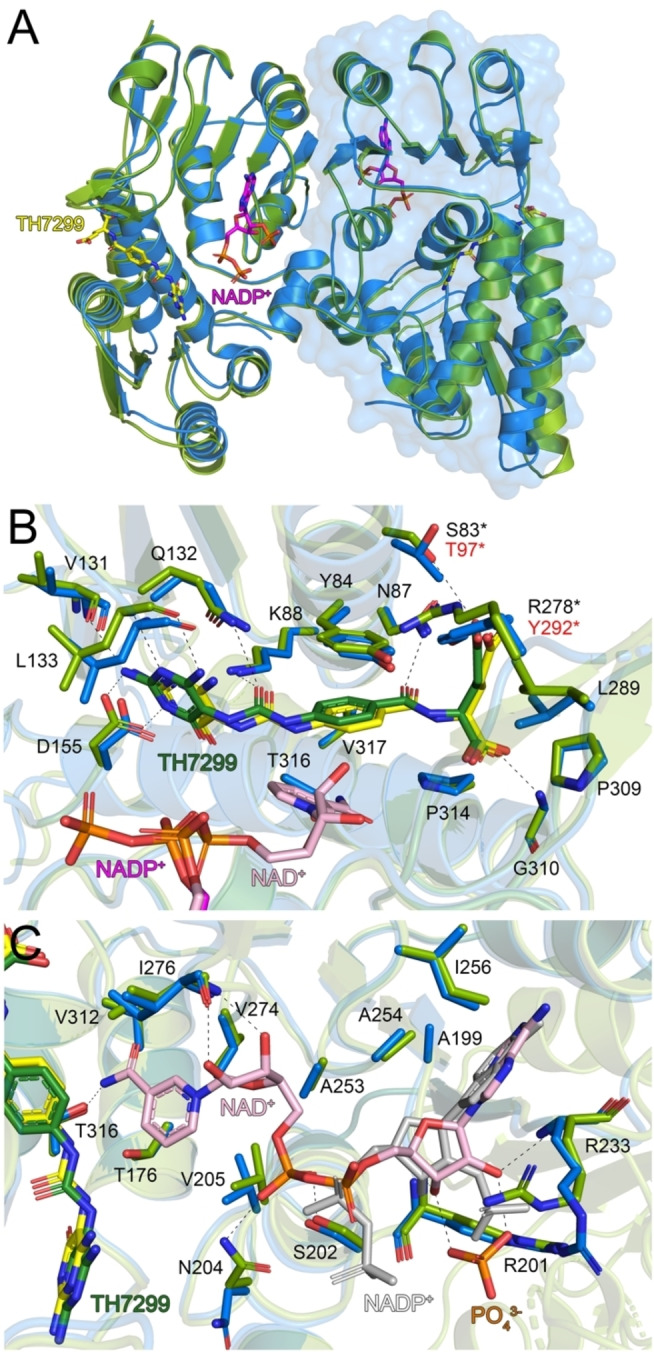
**Comparison of MTHFD2L and MTHFD2** A) Superposition of the hMTHFD2L (blue) and MTHFD2 (green, PDB ID: 6S4E) dimers. One monomer of MTHFD2L is shown as a blue surface representation. The TH7299 inhibitor and partial NADP^+^ cofactor ligands from MTHFD2L are depicted as sticks; C atoms colored yellow (TH7299) or magenta (NADP^+^), O atoms red, N atoms dark blue and P atoms orange. Hydrogen bond networks for B) TH7299 (dark green) in the folate binding site and C) NAD^+^ (light pink) in the co‐factor binding site of MTHFD2. In panel C, NADP^+^ from MTHFD2L is shown as grey sticks. Amino acid numbering corresponds to the MTHFD2 structure. Hydrogen bond interactions are shown as dashed lines. Figures were produced with PyMOL (v.2.3.3, Schrödinger).

MTHFD2L is structurally similar to hMTHFD2 (72 % amino acid sequence identity) and the DC domain of hMTHFD1 (40 % amino acid sequence identity), reflected by the low RMSD values of 0.770 and 1.172 Å, respectively, when the Cα‐atoms of the individual monomers are superimposed. Superposition of our MTHFD2L−TH7299 structure with the MTHFD2‐TH7299 dimer shows the overall structures superimpose very well, with small differences in the positions of two surface‐exposed alpha helices (α1 and α3) (Figure 4A). Detailed comparison of the folate binding sites indicates that TH7299 occupies an almost identical position in both structures (Figure 4B). The residues that interact with TH7299 are nearly identical in both isozymes, an important exception being Arg278 (MTHFD2) which is a tyrosine (Tyr292) in MTHFD2L. This change results in a hydrogen bond between the glutamate tail and the tyrosine in MTHFD2L which is absent in the MTHFD2 structure (Figure 4B).

Furthermore, the absence of a hydrogen bond at this position in MTHFD2 results in the end of the glutamate tail shifting slightly, so that a weaker bond is instead formed with the residue Ser83 (MTHFD2), which is not observed in our MTHFD2L structure, where the equivalent residue is a threonine (Thr97) (Figure 4B). However, as serine and threonine have conserved physicochemical properties this interaction would also be possible in MTHFD2L if Tyr292 was not directing the glutamate tail out of binding range of Thr97. As the glutamate tail (also present in the natural folate substrate) is directed towards the surface of MTFHD2/2L, this part of TH7299 is more flexible compared to the diaminopyrimidine head group and central phenyl ring which are positioned deeper into the binding pocket and are supported by more extensive interactions. The hydrogen bond formed by Tyr292 of MTHFD2L and TH7299 may allow the inhibitor to be positioned more tightly in the folate binding site relative to MTHFD2, explaining the more potent TH7299 inhibition observed for the MTHFD2L isoform.

**Figure 5:**
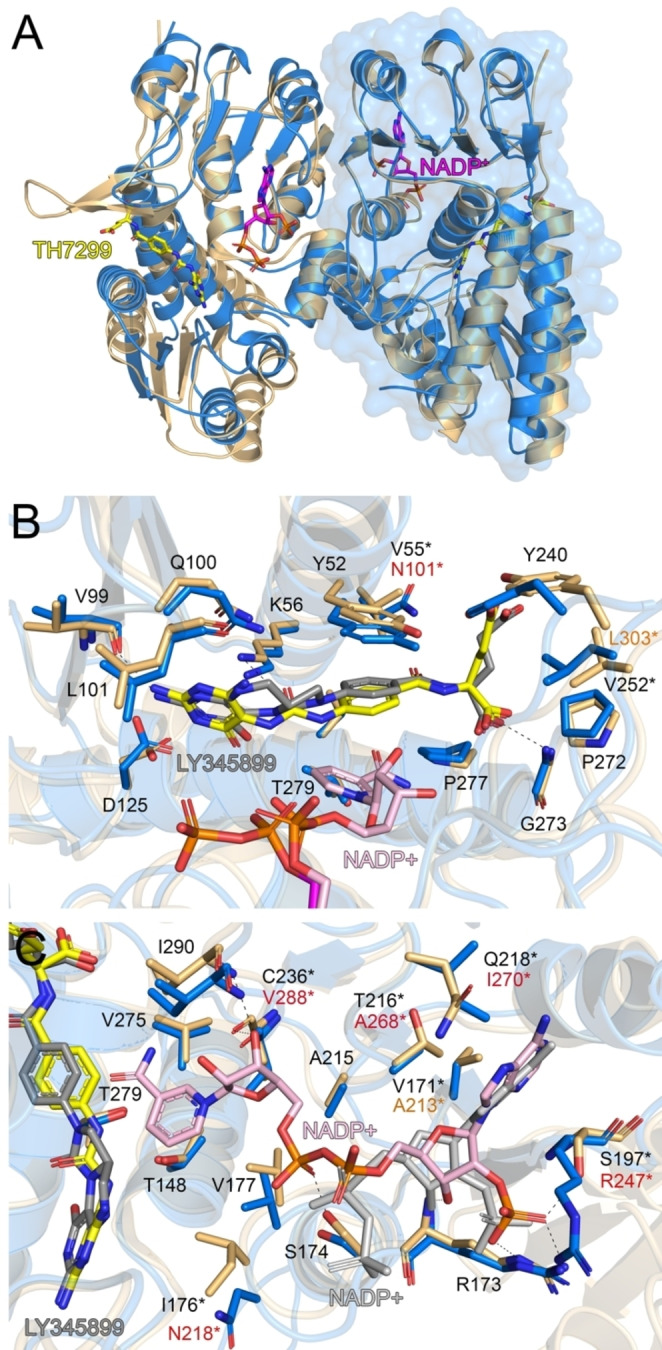
**Comparison of MTHFD2L and MTHFD1−DC** A) Superposition of the hMTHFD2L (blue) and the DC domain of MTHFD1 (light orange, PDB ID: 1DIB) dimers. One monomer of MTHFD2L is shown as a blue surface representation. The TH7299 inhibitor and partial NADP^+^ cofactor ligands from MTHFD2L are depicted as sticks; C atoms colored yellow (TH7299) or magenta (NADP^+^), O atoms red, N atoms dark blue and P atoms orange. Hydrogen bond networks for B) LY345899 (dark grey) in the folate binding site and C) NADP^+^ (light pink) in the co‐factor binding site of MTHFD1−DC. In panel C, partial NADP^+^ from MTHFD2L is shown as grey sticks. Amino acid numbering corresponds to the MTHFD1−DC structure. Hydrogen bond interactions are shown as dashed lines. Figures were produced with PyMOL (v.2.3.3, Schrödinger).

Superposition of MTHFD2L−TH7299 with the dimer of MTHFD1−DC in complex with the inhibitor LY345899 shows the overall structure of MTHFD2L to superimpose less well compared to MTHFD2, with significant structural deviations in four surface‐exposed alpha helices (α1–4) (Figure 5A). Analysis of the folate binding sites shows that both inhibitors superimpose quite well despite LY345899 possessing a bulky tricyclic core compared to the smaller diaminopyrimidine head group of TH7299. The amino acids that surround the inhibitors are highly conserved in both isozymes, with the exceptions of Asn101 and Leu303 (MTHFD2L) which are Val55 and Val252 in MTHFD1 (Figure 5B). In the MTHFD2 structure the equivalent residues are the same as MTHFD2L (Figure 4B).

Assuming a similar binding mode of TH7299 in MTHFD1, this would result in the absence of a hydrogen bond with the carbonyl oxygen between the central phenyl ring and the glutamate tail of TH7299, which is formed with Asn101 in MTHFD2. As MTHFD1 is more strongly inhibited by TH7299 compared to MTHFD2L it implies that the loss of the hydrogen bond may not be so important, and a valine compared to asparagine at this position may provide a more favorable interaction with the hydrophobic phenyl ring of the inhibitor. Regarding the second difference, a valine rather than leucine in close proximity to the glutamate tail of TH7299 may be preferable, as valine is smaller and therefore less hydrophobic, which may be more desirable considering the glutamate tail is flexible and negatively charged at physiological pH. Interestingly, Tyr292 in MTHFD2L (which interacts with the glutamate tail of TH7299) is also a tyrosine (Tyr240) in MTHFD1. As TH7299 inhibits MTHFD1 and MTHD2L significantly stronger than MTHFD2, this further supports the notion that a tyrosine at this position results in enhanced enzyme inhibition by TH7299. As MTHFD1 is more strongly inhibited by TH7299 than MTHFD2L, it implies that the additional inhibition is likely a result of the two aforementioned valine residues (Val55 and Val252) in MTHFD1, since these are the only differences between the isoforms that are relevant for TH7299 binding.

To date, the majority of MTHFD2 inhibitors have been designed to target the folate binding site.[^39^, ^41^] As is evident in our structural analysis, the main differences between the isoforms in the folate binding pocket are in residues that are positioned more towards the surface of the protein, rather than deep within the binding cavity, where the residues are identical. Clearly, this makes the development of truly isoform‐selective MTHFD2 inhibitors challenging. While it was reported that a series of sulfonamide inhibitors were potent and selective for MTHFD2 over MTHFD1−DC,^[39]^ follow‐up studies indicated that the compounds were significantly less potent and did not display high MTHFD2/MTHFD1 selectivity.^[41]^ In our previous work, we modified TH7299 to produce significantly more potent MTHFD2 inhibitors (TH9619 and TH9028), both of which were still more active against MTHFD1−DC than MTHFD2.^[41]^ Recently, MTHFD2 inhibitors targeting a newly discovered allosteric site were shown to be MTHFD2/MTHFD1‐selective, however it should be noted that inhibition towards MTHFD2L was not reported in the study.^[40]^

### Comparison of MTHFD2L with related isoforms: cofactor binding site

Superposition of the cofactor binding sites of MTHFD2 and MTHFD2L indicates that all of the residues involved in NAD^+^/NADP^+^ binding are identical (Figure 4C). In contrast, comparison with MTHFD1 shows that there are six amino acids involved in cofactor binding that are not conserved between the structures (Figure 5C). Specifically, the residues Val171, Ile176, Thr216, Gln218, Ser197 and Cys236 in MTHFD1 are Ala213, Asn218, Ala268, Ile270, Arg247 and Val288 in MTHFD2L (Figure 5C). Notably, five out of six of these differences have non‐conserved physicochemical properties. To date, no MTHFD2 inhibitors targeting the cofactor binding site exist. While there are clear differences between MTHFD2 and MTHFD1 which could aid in the design of compounds with MTHFD2/MTHFD1 selectivity, the identical cofactor binding sites of MTHFD2 and MTHFD2L makes achieving MTHFD2/MTHFD2L selectivity virtually impossible. It is therefore evident that targeting the cofactor binding site is clearly less useful compared to the folate binding or allosteric sites of MTHFD2.

### Comparison of MTHFD2L with related isoforms: allosteric site

**Figure 6:**
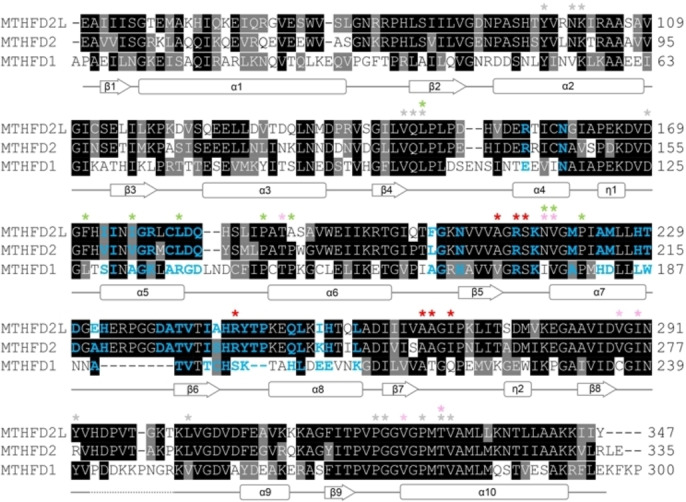
**Structure‐based sequence alignment of hMTHFD2L with closely related isozymes hMTHFD2 and hMTHFD1−DC**. Amino acid sequence comparison of human MTHFD2L (UniProt: Q9H903), human MTHFD2 (UniProt: P13995) and the human MTHFD1−DC domain (UniProt: P11586, residues 1–300) performed using Clustal Omega through the EBI webserver. The transit peptides (residues 1–35) of the mitochondrial isoforms MTHFD2L and MTHFD2 are not shown. The resulting alignment is colored according to sequence similarity using BOXSHADE. Identical residues are shaded black, while grey shading indicates amino acids with conserved physicochemical properties. Residues in hMTHFD2L which interact with the inhibitor TH7299 in the folate binding site are indicated by grey asterisks. Residues that interact with partial NADP^+^ in hMTHFD2L−TH7299 in the cofactor binding site are indicated by red asterisks and additional residues that interact with NAD^+^ in hMTHFD2−TH7299 (PDB ID: 6S4E) are shown as pink asterisks. Amino acids that interact with the ligand J4C in hMTHFD2 (PDB ID: 7EHM) in the allosteric site are shown as green asterisks. The secondary structure corresponding to the amino acid sequence of hMTHFD2L−TH7299 is displayed below the alignment. The segment of missing density in this structure is indicated by a dotted line. Residues in the MTHFD2L dimer interface and the equivalent residues in MTHFD2 and MTHFD1−DC are shown as bold blue letters.

**Figure 7:**
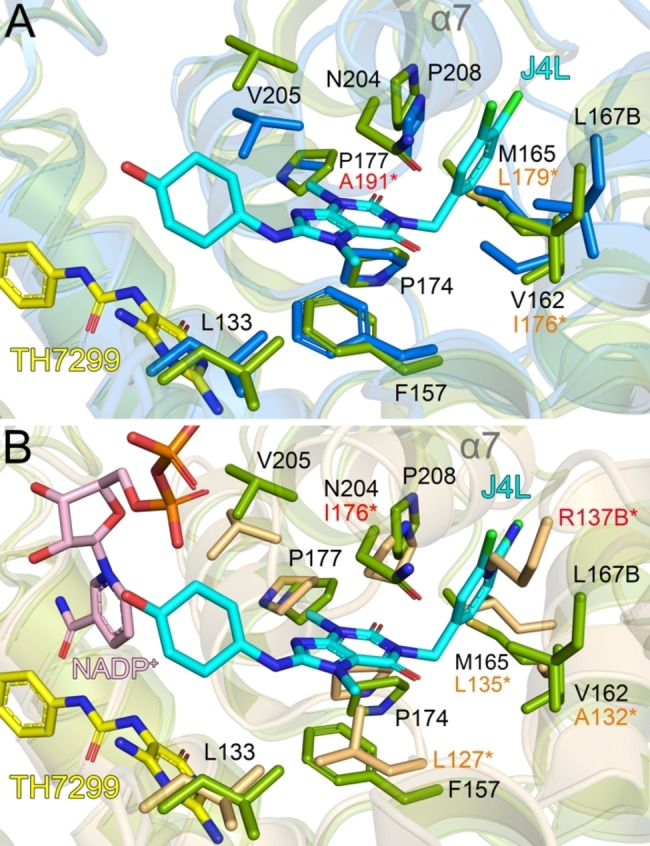
**Comparison of the MTHFD2 allosteric site with MTHFD2L and MTHFD1−DC** A) Superposition of MTHFD2 (green, PDB ID: 7EHV) with A) MTHFD2L (blue) and B) MTHFD1−DC (light orange, PDB ID: 1DIB). The allosteric inhibitor J4L from MTHFD2, the folate binding site inhibitor TH7299 from MTHFD2L and the NADP^+^ cofactor from MTHFD1−DC are depicted as sticks; C atoms colored cyan (J4L), yellow (TH7299) or light pink (NADP^+^), O atoms red, N atoms blue, Cl atoms green and P atoms orange. Amino acid numbering corresponds to the MTHFD2 structure. Alpha helix 7 (α7) which moves upon J4L binding is highlighted. Residues N218 (MTHFD2L) and I176 (MTHFD1−DC) from α7, as well as partial NADP^+^ from MTHFD2L are not shown for clarity as they directly clash with J4L. NAD^+^ is not bound in the MTHFD2J4L structure as the allosteric inhibitor directly interferes with cofactor binding. Figures were produced with PyMOL (v.2.3.3, Schrödinger).

Recently, xanthine derivatives were reported to inhibit MTHFD2 though binding to an allosteric site, distinct from the folate or cofactor binding sites. It was shown that conformational changes occurred upon inhibitor binding, which were proposed to impede cofactor and phosphate binding to MTHFD2. The inhibitors were confirmed to inhibit MTHFD2 in a non‐competitive manner and were reported to be more active towards MTHFD2 (IC_50_: >0.78 μM) than MTHFD1 (IC_50_: >10 μM).^[40]^ Comparison of the binding mode of the allosteric MTHFD2 inhibitor J4L with MTHFD2L indicates that there are three differences between the structures. Specifically, Pro177, Met165 and Val162 in MTHFD2 are Ala191, Ile176 and Leu181 in MTHFD2L (Figures 6 and 7A). These residues have conserved physicochemical properties.

Comparison of the MTHFD2 allosteric site with MTHFD1 shows that there are five differences between the isoforms. This includes Phe157, Asn204, Met165, Val162 and Leu167* (*denotes residue is from the second monomer) in MTHFD2, which are Leu127, Ile176, Leu135, Ala132 and Arg137* in MTHFD1 (Figures 6 and 7B). Of these residues, three have conserved physicochemical properties. However, it should be noted that while both Phe157 (MTHFD2) and Leu127 (MTHFD1) are both hydrophobic amino acids, having an aromatic residue at this position is particularly important, as in MTHFD2 Phe157 is known to form a pi‐stacking interaction with the xanthine core of the inhibitor J4L.^[40]^ Such an interaction is not present in MTHFD1 (Figure 7B), which is likely a reason for the less potent inhibition towards this isoform. However, MTHFD2L also has a phenylalanine (Phe171) at this position so the pi‐stacking interaction with J4L would be maintained (Figure 7A).

Clearly the allosteric site has a lot of potential for the design of selective MTHFD2/MTHFD1 inhibitors due to important differences between the isoforms. The development of MTHFD2/MTHFD2L‐selective inhibitors is clearly more challenging as there are no clear differences in this part of the allosteric binding pocket. While the differences between the allosteric sites of MTHFD2 and MTHFD2L are physicochemically conserved, the inhibition properties are still unlikely to be identical. It would certainly be of interest to determine whether MTHFD2L is inhibited more strongly or weakly by these newly discovered allosteric inhibitors.

### Comparison of MTHFD2L with related isoforms: dimer interface

**Figure 8:**
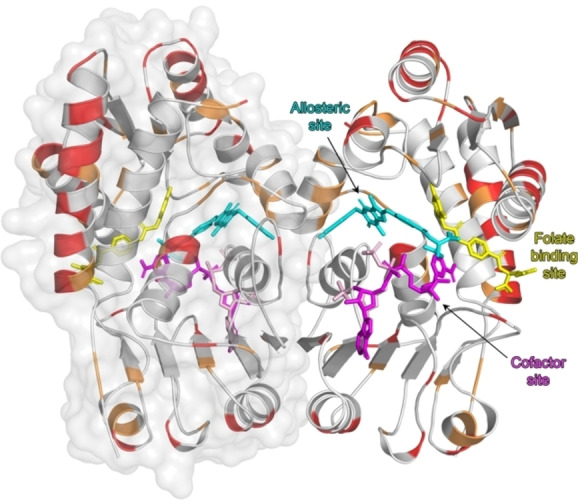
**Structure of the hMTHFD2L dimer highlighting the three different ligand binding sites**. The surface of one monomer is shown at 80 % transparency. The structure was superimposed with that of MTHFD2−TH7299 (PDB ID: 6S4E) and MTHFD2−J4C (PDB ID: 7EHM), however only the ligands of the compared structures are shown. TH7299 which binds in the MTHFD2L folate binding site is shown as yellow sticks. Partial NADP^+^ (MTHFD2L) and NAD^+^ (MTHFD2) which bind in the cofactor binding site are shown as light pink and magenta sticks, respectively. The allosteric inhibitor J4C (MTHFD2) is shown as cyan sticks. Amino acid differences between MTHFD2L and MTHFD2 are highlighted. Red coloring indicates non‐conserved differences and orange coloring indicates differences with conserved physicochemical properties. Figures were produced with PyMOL (v.2.3.3, Schrödinger).

Analysis of the dimer interface in terms the folate, allosteric and co‐factor binding sites indicates this area does play a small role in ligand binding for two of the sites (Figures 6 and 8). There are three residues (Arg247, Arg215 and His246, MTHFD2L numbering) in the dimer interface which interact with NADP^+^ (MTHFD2L) or NAD^+^ (MTHFD2) in the cofactor binding site and two residues that interact with the inhibitor J4L in the allosteric site (Ile176 and Leu181, MTHFD2L numbering). The aforementioned residues are identical between MTHFD2L and MTHFD2, with the exception of Ile176 which is a valine (Val162) in MTHFD2. There are no residues from the dimer interface that are responsible for binding TH7299 in the folate binding site (Figure 6). It has been proposed that disrupting the dimer interface may be a good strategy for the development of more selective MTHFD2 inhibitors.^[38]^ Comparison of the dimer interfaces of MTHFD2L and MTHFD2 indicates that 87.5 % of the residues are identical, 7.5 % have conserved physicochemical properties and only 5 % are non‐conserved. In contrast, when the MTHFD2L dimer interface is compared to MTHFD1−DC only 25 % of the residues are identical, 5 % are physicochemically conserved and 70 % are non‐conserved (Figure 6). This indicates that while the dimer interface may be a reasonable target for developing MTHFD2/MTHFD1‐selective inhibitors as there are significant differences between the isoforms, it is clearly a far less useful target for the development of MTHFD2/MTHFD2L selective inhibitors.

A limiting factor of clinically used antimetabolite drugs such as methotrexate and pemetrexed that target other 1C‐metabolism enzymes,^[51]^ is that they also target non‐cancerous cells which can result in severe side‐effects such as bone marrow toxicity and cardiac abnormalities during treatment.[^52^, ^53^, ^54^] The structural and sequence analysis presented here indicates that the majority of differences between MTHFD2 and MTHFD2L are located in surface exposed areas of the proteins, rather than within the three ligand binding pockets (Figure 8). Clearly, MTHFD1/MTHFD2 inhibitor selectivity is far more feasible than the development of highly specific MTHFD2/MTHFD2L inhibitors. This is problematic as non‐isoform‐selective MTHFD2 inhibitors would also target healthy cells. However, it should be noted that a general MTHFD‐targeted inhibitor may have certain advantages. Specifically, while RNAi silencing of *MTHFD2* has shown that cancer cell growth is dependent on MTHFD2,[^34^, ^41^, ^55^] this contrasts with studies of viable CRISPR‐Cas‐9 mediated *MTHFD2* knockout cells.^[56]^ Such viability may be a result of the high plasticity of the 1C‐metabolism pathway, and that under certain conditions, such as survival selection pressure upon *MTHFD2* deletion, the mitochondrial and cytoplasmic pathways are able to compensate for one another.^[57]^ For example, it has been shown that the normally non‐essential cytoplasmic 1C‐metabolism enzyme SHMT1 is essential in MTHFD2 knockout cells.^[56]^ Therefore, MTHFD2 inhibitors that also target MTHFD1 and MTHFD2L maybe be beneficial for counteracting emerging resistance, should it arise.

## Conclusion

In recent years MTHFD2 has emerged as an attractive anticancer drug target as it is upregulated in cancer but is virtually absent in healthy adult cells.[^10^, ^30^, ^33^, ^34^] Selective targeting of MTHFD2 could result in anticancer therapies with reduced side‐effects. However, the existence of the closely related isoforms cytoplasmic MTHFD1 and mitochondrial MTHFD2L, which are involved in the 1C‐metabolism pathway in healthy cells, makes this very challenging. A limitation of studies aimed at developing selective MTHFD2 inhibitors is that they often only focus on comparisons with MTHFD1 and ignore MTHFD2L inhibition.[^39^, ^40^] This is particularly relevant as the majority of 1C‐units used in cytoplasm are in fact produced in the mitochondria.[^4^, ^5^, ^6^] It has recently been shown that one of most potent MTFHD2 inhibitors reported to date, TH7299, is actually a more potent inhibitor of both MTHFD2L and MTHFD1.^[41]^ We have solved the first structure of human MTHFD2L and its complex with the inhibitor TH7299, which revealed an interaction between a tyrosine residue and the glutamate moiety of the inhibitor, which is also present at the same position in MTHFD1. This residue is an arginine in MTHFD2 and so this difference is likely important for the enhanced inhibition of TH7299 towards MTHFD1 and MTHFD2L isoforms. Detailed structural comparison of MTHFD2L with MTHFD2 indicates that the majority of differences between the enzymes are in surface‐exposed regions and that the folate, cofactor and allosteric sites in addition to the dimer interface, show very high structural conservation. In contrast, MTHFD2L and MTHFD2 show significant differences with MTHFD1 in the three ligand binding sites as well as the dimer interface. Overall, this indicates that the development of MTHFD2/MTHFD2L‐selective inhibitors is significantly more challenging than the development of MTHFD2/MTHFD1‐selective inhibitors. The MTHFD2L structure presented here will be a useful tool for future studies towards the development of selective MTHFD inhibitors.

## Experimental Section

### Protein overexpression and purification

An expression construct of human MTHFD2L (corresponding to amino acids 50–347) was prepared in pET22b (Novagen). His‐tagged hMTHFD2L was expressed in *E. coli* Arctic Express (DE3) competent cells overnight at 13 °C. The cells were harvested and lysed using an Emulsiflex C3 Homogenizer. MTHFD2L protein was then purified using a HiTrap TALON column (GE Healthcare) followed by Size‐exclusion chromatography using a HiLoad 16/60 Superdex75 column (GE Healthcare). Following purification the protein was exchanged into storage buffer (50 mM Tris−HCl pH 8.0, 100 mM KCl, 5 % (v/v) glycerol, 1 mM TCEP) using a PD‐10 column (GE Healthcare).

### Synthesis of TH7299

TH7299 was synthesized as reported previously.^[41]^

### Crystallization

Purified MTHFD2L (7.5 mg/mL) was preincubated with 2 mM TH7299 and 5 mM NADP^+^ for 45 minutes on ice. The protein was crystallized via sitting drop vapor diffusion with 0.2 M NaF, 20 % (w/v) PEG3350 at 20 °C. MTHFD2L crystals formed after 3 weeks were added briefly to a cryoprotectant solution consisting of the respective growth condition supplemented with 25 % (v/v) glycerol, before being flash frozen in liquid nitrogen.

### Data collection, structure determination, and refinement

X‐ray diffraction data was collected at beamline PXII of the Swiss Light Source (Villigen, Switzerland) equipped with a PILATUS‐2MF detector. A complete dataset was collected using a single crystal at a wavelength of 1.00 Å. The data was processed and scaled with XDS^[58]^ and Aimless^[59]^ within the CCP4 suite.^[60]^ Molecular replacement was performed in Phaser^[61]^ using the structure of human MTHFD2 (PDB ID: 5TC4) with ligands and waters removed, as the search model. Several cycles of model building and refinement were performed using Coot^[62]^ and Refmac5^[63]^ during which waters and ligands were added to the structure. Data processing and refinement statistics are presented in Table 1. The coordinates and structure factors for hMTHFD2L were deposited in the PDB under the accession code 7QEI.

### MTHFD2L inhibition assay

MTHFD2L activity was monitored using the NAD(P) Glo™ Detection System (Promega). The assay monitors the concentration of NADPH and in the presence of this reduced cofactor the enzyme Reductase reduces a proluciferin reductase substrate to form luciferin, which can then be quantified using Ultra‐Glo recombinant Luciferase. The luminescence produced is proportional to the amount of NADPH in the sample. The assays were performed according to the manufacturers instructions. Each reaction consisted of assay buffer (50 mM Tris−HCl pH 8.0, 100 mM KCl, 5 mM MgCl_2_, 2 mM 2‐Mercaptoethanol, 5 % (v/v) Glycerol, and 0.005 % (v/v) Tween 20) using 100 nM MTHFD2L, 100 μM NADP^+^ (Sigma, Cat # N5755) and 40 μM Folitixorin (Toronto Research Chemicals, Cat # F680350). Assays were performed using white non‐binding surface 384‐well assay plates (Corning, Cat # 3824). Wells where MTHFD2L was omitted were included on the assay plate as a background control. The IC_50_ value for TH7299 was determined from an 11‐point dose‐response curve generated using an acoustic dispenser (Labcyte, Echo 550 Liquid handler). Assay points were run in duplicate and DMSO was used as negative control. The IC_50_ value was determined using nonlinear regression by fitting the curve log[inhibitor] vs. response ‐ Variable slope (four parameters) to the data using GraphPad Prism 6.0 software (La Jolla California, USA).

## Conflict of interest

Thomas Helleday, Martin Henriksson, Evert Homan and Pål Stenmark are named inventors on patent application PCT/EP2019/059919. MTHFD2 inhibitors are developed toward the clinic by the company One‐Carbon Therapeutics AB, currently owned by The Helleday Foundation (THF), a not‐for‐profit charitable foundation. Thomas Helleday is a board member of THF. The remaining authors declare no competing interests.

1

## Data Availability

The data that support the findings of this study are available from the corresponding author upon reasonable request.
